# Oxychloride of Zinc Treatment of Dental Pulps

**Published:** 1871-07

**Authors:** H. A. Smith


### ARTICLE VIII.

### *Oxychloride of Zinc Treatment of Dental Pulps.*

By H. A. Smith, D.D.S

Recognizing, as nearly all do, that the dental pulp is the proper organ through which the teeth receive their nourishment, and that therefore there can be no real safety to the tooth without the presence of the pulp in its normal condition, it is our duty as conscientious practitioners of dentistry, to preserve this organ whenever in our power.

That the dental pulp can, under favorable circumstances, be kept so as to perform its proper physiological functions after exposure from caries or other cause, is now generally admitted.

The methods of treatment recommended to accomplish this end, have been as numerous as some of them were novel, from the early impracticable method of bridging or arching over the pulp with gold foil. The innumerable non-conducting materials suggested from time to time, on down to the surgical method of gouging out a portion of the pulp, each in turn have been tried and found lacking. But dental science advances, and it remains to us who live in these latter days, to avail ourselves of the modern discovery of a *panacea* for all the ills of exposed or diseased dental pulps.

Strange as the claims made for some of the older methods may appear to us now, *none* perhaps have had a degree of success claimed in their use at all to be compared to the oxychloride of zinc treatment.

A collection indeed, of the discussions and papers published relating to treating and capping exposed dental pulps during the last ten years, would present a most curious array of confident statements, upon the least positive evidence, as to the statistics or facts relating to the subject that could be found in any department of modern science.

If we may judge from the statements of the few who have made public the results of treatment with the oxychloride, the same idiocyncrasy to claim success without giving the data upon which to base them, is still to be observed. It will require years of carefully recorded experience to es tablish what is already claimed for this method.

Oxychloride of zinc has most of the requisite qualities, that in our judgement, are desirable in a material used as a capping for exposed pulps. It is sufficiently indestructible when protected from the secretions of the mouth. Its conductivity is low, thus preventing disturbance to the pulp by thermal changes. It is dense and hard enough to resist the necessary pressure to complete the operation of filling. It has the requisite plasticity to be applied directly upon the denuded pulp, forming a close fitting cap without crowding upon it.

One other quality which we have ever regarded as indispensable—it lacks, viz: that the material should have no chemical or irritant effects when in contact with the pulp tissue.

Oxychloride of zinc is an escharotic and an irritant. It has been asserted, that it is this chemical action upon the pulp tissue which renders it specially applicable for this purpose.

To this position we must take exceptions. A pulp in its normal state of health, if an exposed pulp may be so regarded, should be let alone as nearly as possible. The irritant effect of oxychloride of zinc causes a departure from this healthy state, which would render success much less certain than if the capping was entirely passive.

A favorite method of treatment has been to cauterize the point of exposure with creosote before applying the capping, thus preventing the full effects of the escharotic action, and destroying in part this property which it is thought recommends the oxychloride.

If the pulp is diseased from long exposure or the pres ence of irritating matter in contact with it, and treatment is desirable, then the specific action of either the creosote or chloride of zinc may be indicated, but they should be applied as therapeutic agents. The remedy used is more fully under our control when applied for a definite object, than when combined with an agent with which we hope to effect a double purpose.

The application of an agent that would not prove an irritant to the pulp, when it is in a proper condition to be covered over, to prevent the pain caused by theoxychloride is very desirable, and has been eagerly sought after. The creosote partially effects this, but it is also an irritant, and unless the case indicates it, should not be applied. It alters the structure of the pulp tissue, forming a coagulum or pellicle which must be absorbed, or remain an irritant or foreign body in contact with it, which would greatly endanger the vitality of the pulp.

It would appear then, that those of the profession who recommend the oxychloride of zinc as a capping, for the reason of its escharotic action upon the pulp, depart from their own theory, when they advise that an agent be first applied to counteract the legitimate effects of the chloride.

What we need is a substance, a compound having all the properties we have enumerated, that shall be received in contact with the pulp tissue, without producing irritation. save that which may follow from contact with any foreign body, no matter what its properties are.—*Dental Register.*

---

